# 

**Published:** 1854-07

**Authors:** 


### WESTERN JOURNAL

OF

### MEDICINE AND SURGERY.

NEW SERIES. JULY, 1854. VOL. II.—NO. 7.

EDITED BY

LUNSFORD P. YANDELL, M. D.

PROFESSOR OF PHYSIOLOGY AND PATHOLOGICAL ANATOMY, IN THE UNIVERSITY OF LOUISVILLE.

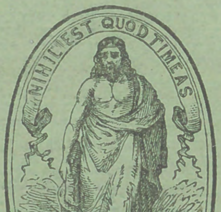

Surgeon Genl's Office
LIBRARY.
Washington, D.C.

LOUISVILLE, KY:

PRINTED AND PUBLISHED BY J. F. BRENNAN,

*Cor. of Market & First Streets.*

PRICE, $3 A YEAR—INVARIABLY IN ADVANCE.—POSTAGE, 2 CENTS A NUMBER.

